# mTOR Pathway in Papillary Thyroid Carcinoma: Different Contributions of mTORC1 and mTORC2 Complexes for Tumor Behavior and *SLC5A5* mRNA Expression

**DOI:** 10.3390/ijms19051448

**Published:** 2018-05-13

**Authors:** Catarina Tavares, Catarina Eloy, Miguel Melo, Adriana Gaspar da Rocha, Ana Pestana, Rui Batista, Luciana Bueno Ferreira, Elisabete Rios, Manuel Sobrinho Simões, Paula Soares

**Affiliations:** 1 Instituto de Investigação e Inovação em Saúde (i3S), Universidade do Porto, Porto 4099-002, Portugal; ctavares@ipatimup.pt (C.T.); celoy@ipatimup.pt (C.E.); jmiguelmelo@live.com.pt (M.M.); adrianar@ipatimup.pt (A.G.d.R.); apestana@ipatimup.pt (A.P.); rbatista@ipatimup.pt (R.B.); lferreira@ipatimup.pt (L.B.F.); erios@ipatimup.pt (E.R.); ssimoes@ipatimup.pt (M.S.S.); 2 Institute of Molecular Pathology and Immunology of the University of Porto (IPATIMUP), Porto 4099-002, Portugal; 3 Faculty of Medicine, Porto University, Porto 4099-002, Portugal; 4 Department of Endocrinology, Diabetes and Metabolism, University and Hospital Center of Coimbra, 3000-075 Coimbra, Portugal; 5 Faculty of Medicine, University of Coimbra, Coimbra 3004-504, Portugal; 6 Public Health Unit, ACeS Baixo Mondego, Coimbra 3000-075, Portugal; 7 Programa de Oncobiologia Celular e Molecular, Instituto Nacional de Câncer, Rio de Janeiro 20230-130, Brasil; 8 Department of Pathology, Medical Faculty of the University of Porto, Porto 4099-002, Portugal; 9 Department of Pathology, Hospital de S. João, Porto 4200-319, Portugal

**Keywords:** mTOR, thyroid cancer, sodium iodide symporter (NIS)/*SLC5A5*

## Abstract

The mammalian target of rapamycin (mTOR) pathway is overactivated in thyroid cancer (TC). We previously demonstrated that phospho-mTOR expression is associated with tumor aggressiveness, therapy resistance, and lower mRNA expression of *SLC5A5* in papillary thyroid carcinoma (PTC), while phospho-S6 (mTORC1 effector) expression was associated with less aggressive clinicopathological features. The distinct behavior of the two markers led us to hypothesize that mTOR activation may be contributing to a preferential activation of the mTORC2 complex. To approach this question, we performed immunohistochemistry for phospho-AKT Ser473 (mTORC2 effector) in a series of 182 PTCs previously characterized for phospho-mTOR and phospho-S6 expression. We evaluated the impact of each mTOR complex on *SLC5A5* mRNA expression by treating cell lines with RAD001 (mTORC1 blocker) and Torin2 (mTORC1 and mTORC2 blocker). Phospho-AKT Ser473 expression was positively correlated with phospho-mTOR expression. Nuclear expression of phospho-AKT Ser473 was significantly associated with the presence of distant metastases. Treatment of cell lines with RAD001 did not increase *SLC5A5* mRNA levels, whereas Torin2 caused a ~6 fold increase in *SLC5A5* mRNA expression in the TPC1 cell line. In PTC, phospho-mTOR activation may lead to the activation of the mTORC2 complex. Its downstream effector, phospho-AKT Ser473, may be implicated in distant metastization, therapy resistance, and downregulation of *SLC5A5* mRNA expression.

## 1. Introduction

Thyroid cancer (TC) is the most common endocrine neoplasia. Differentiated thyroid carcinoma (DTC) arises from thyroid follicular cells and represents more than 90% of all cases of TC. DTC comprises papillary thyroid carcinoma (PTC) and follicular thyroid carcinoma (FTC), PTC being the most prevalent type [1,2]. PTC can be further subdivided into the so-called classic PTC (cPTC) and the follicular variant of PTC (fvPTC) [1]. 

PTC carries an overall good, or even very good, prognosis (overall survival rates of >95% at 25 years) [3] after being treated with surgery followed by radioactive iodine (RAI) therapy at adequate levels [4]. Due to poorly understood reasons, a subgroup of TC patients (10–15%) become resistant to RAI treatment [4], leading to recurrence and/or metastization and a significant reduction of 10-year survival rate [5,6]. The molecular mechanism behind this resistance relies, at least in part, on the loss of sodium iodide symporter (NIS) expression and/or function. NIS is codified by the *SLC5A5* (Solute Carrier Family 5 Member 5) gene, being normally expressed in the basolateral membrane of thyroid follicular cells. It is the gateway of iodine into the interior of the follicular cells as it becomes further incorporated in thyroid hormones. Usually, PTCs maintain NIS expression and function allowing the incorporation of ^131^I causing tumor cell death, a very efficient targeted radiotherapy [7].

The mTOR pathway can be activated by diverse stimuli, such as growth factors, nutrients, energy, stress signals, and other essential signaling pathways, such as phosphoinositide 3-kinase (PI3K) and mitogen-activated protein kinase (MAPK) [8,9,10]. It is overactivated in a variety of human neoplasms [10], including TC [9,11,12]. mTOR can associate with distinct proteins and form two distinct complexes: mTORC1 and mTORC2. The complexes have different downstream effectors and physiological functions: mTORC1 effectors are S6K1 and 4EBP1, which participate in cellular growth, proliferation, and survival, whereas mTORC2 can phosphorylate protein kinase C-α (PKC-α) and AKT (Ser 473) and regulates the actin cytoskeleton and cell migration [8,10].

A recent study by our group demonstrated that phospho-mTOR is a marker of aggressiveness in PTC; its expression was associated with aggressive clinicopathological features, including distant metastases, resistance to ^131^I therapy and, consequently, worse prognosis [13]. In the same study, we observed that phospho-S6 expression was associated with clinicopathological features of low aggressiveness and we did not find a significant correlation between phospho-mTOR and phospho-S6 expression in the tumors [13]. The absence of correlation between the two proteins and their divergent behavior led us to hypothesize that, in PTC, the activation of phospho-mTOR might be contributing preferentially to the activation of the mTORC2 complex, and consequently to AKT phosphorylation (phospho-AKT Ser473) [13], as it has been observed in other tumor models [14,15,16,17]. Phospho-AKT is upregulated in PTC [9,11,12,18], but its role in PTC clinical behavior and resistance to therapy needs to be further explored.

Previous studies showed that NIS expression increases when the mTOR pathway is inhibited [6], however, such studies only explored the role of mTORC1 complex [19,20]. As far as we are aware, the role of mTORC2 on *SLC5A5* mRNA expression has not been previously analyzed. So far, it is only known that dual inhibition of mTORC1 and mTORC2 complexes by Torin2 in TC models causes a decrease in cell growth [21,22] and inhibits metastization [22]. In this study, we intended to understand the relevance of mTORC2 complex activation in PTC, by exploring the role of phospho-AKT Ser473 in PTC clinical behavior and the response of TC derived cell lines to Torin2 dual inhibition of mTORC1 and mTORC2 complexes.

## 2. Results

### 2.1. Immunoexpression of Phospho-AKT Ser473 in PTC

The expression of phospho-AKT Ser473 was negative in 49.5% of cases. 50.5% of positive cases were distributed throughout the score values (Table 1). In the group of positive cases, immunostaining was detected only in the cytoplasm in 40/92 of cases, and concurrently in the cytoplasm and nucleus in 52/92 of cases. 

**Figure 1:**
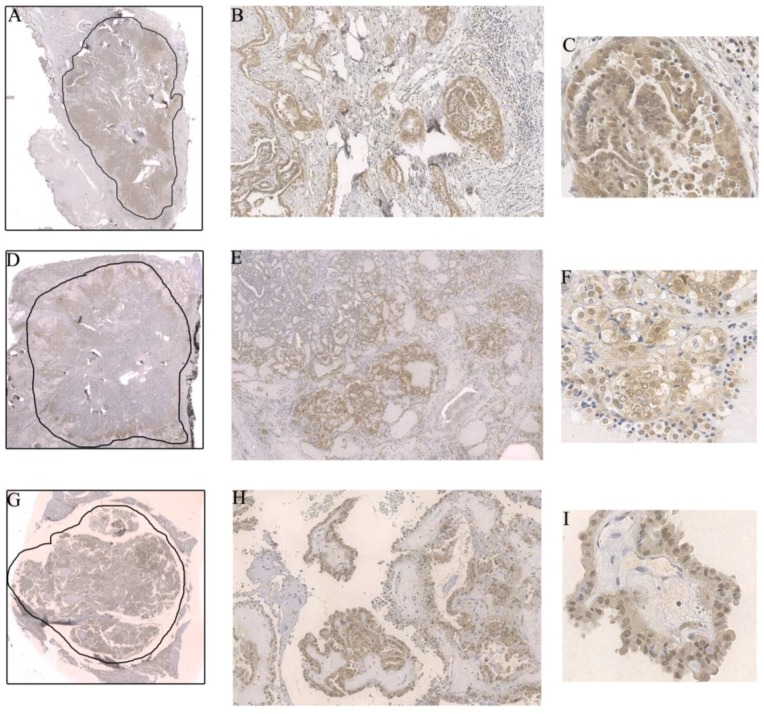
(**A**–**C**) Intensification of the immunostaining and phospho-AKT Ser473 nuclear expression in the invasive front of a classic papillary thyroid carcinoma (cPTC); (A) 0.44×, (B) 10×, and (C) 40× magnification; (**D**–**F**) Preferential phospho-AKT Ser473 expression in the tumor periphery, another example in a cPTC. Notice that, in this case, the nuclear translocation was not so intense compared to the previous one; (D) 0.44×, (E) 4×, and (F) 40× magnification; (**G**–**I**) Strong and disseminated phospho-AKT Ser473 nuclear expression in a hobnail variant of papillary thyroid carcinoma (PTC); (G) 0.44×, (H) 10×, and (I) 40× magnification. The drawn lines, at 0.44× magnification (Figure 1A,D,G), circumscribe the tumor.

Among the positive cases, phospho-AKT Ser473 was more intense and preferentially located at the invasive front in 44% of the tumors (Figure 1). Once in the tumor’s periphery, phospho-AKT Ser473 was more frequently located in the nucleus (67.6% of the cases with phospho-AKT Ser473 in the invasive areas of the tumor displayed nuclear staining) (Figure 1).

### 2.2. Relationship between the Phospho-AKT Ser473 Expression and Clinicopathological and Molecular Features

Phospho-AKT Ser473 total expression (cytoplasm plus nuclear) was positively correlated with phospho-mTOR expression (r(168) = 0.2, *p* = 0.02) but not with phospho-S6 expression (r(139) = 0.02, *p* = 0.8). 

Phospho-AKT Ser473 was significantly more expressed in PTCs harboring the *BRAF*V600E mutation than in *BRAF* wild type (WT) PTC (*p* = 0.04) (Table 2); when divided by histological variant this significant association was maintained in the cPTC group but was lost in the fvPTC group. There were no significant associations between phospho-AKT Ser473 total expression and the following features: age, tumor size, tumor capsule, multifocality, lymphocytic infiltrate, vascular invasion, lymph node metastases, tumor margins (well circumscribed vs. infiltrative), distant metastases, staging, *NRAS* and *TERT*p status, number of ^131^I therapies or cumulative dose of radioactive iodine, additional treatments, disease-free status at one year, and disease-free status at the end of follow-up.

The nuclear expression of phospho-AKT Ser473 was more often detected in cases with distant metastases compared with cases without distant metastases (*p* = 0.04) (Table 3). We did not find any significant association between phospho-AKT Ser473 nuclear expression and other clinicopathological or molecular features (all PTCs, and cPTC or fvPTC subgroups).

### 2.3. Contribution of mTORC1 and mTORC2 Complexes in the Regulation of SLC5A5 mRNA Expression

To study the role of both mTORC1 and mTORC2 complexes on *SLC5A5* mRNA expression, we performed treatments of the TPC1 and K1 cell lines with RAD001 (mTORC1 inhibitor) and Torin 2 (mTORC1 and mTORC2 dual inhibitor) for 60 and 72 h.

**Figure 2:**
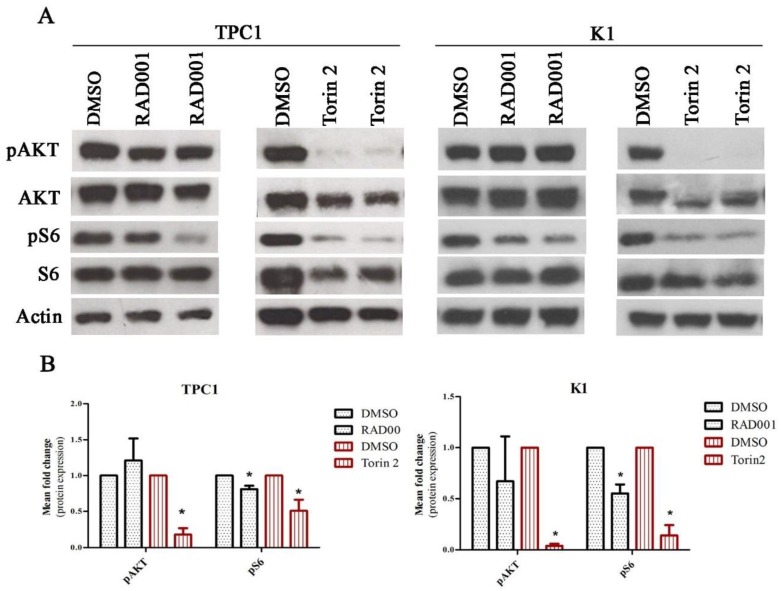
RAD001 and Torin2 effect on TPC1 and K1 cell lines. (**A**) Cells were treated with 20 nM of RAD001 and 450 nM of Torin2 for 72 h. Western blot analysis of RAD001 and Torin2 effect on the activation status of mTORC1 and mTORC2 complexes was evaluated by phospho-S6 Ser235/236 and phospho-AKT Ser473 expression, respectively. Representative actin expression is shown. Protein level in treated cells was evaluated in duplicate. (**B**) Mean fold change of protein expression observed in TPC1 cell line treated with 20 nM of RAD001 and 450 nM of Torin2 in comparison to cells treated with DMSO. Phosphorylated proteins were normalized by the levels of their correspondent total proteins. Results are shown as mean expression value of three independent experiments ±SEM. * *p* < 0.05 (unpaired Student’s *t* test).

We confirmed the efficacy of the drugs in the activity of mTOR complexes through the evaluation of phospho-S6 Ser235/236 expression as a read-out of mTORC1 activity and phospho-AKT Ser473 as read-out of mTORC2 activity. After 72 h of treatment, RAD001 caused a significant downregulation of the mTORC1 complex and did not affect the activity of the mTORC2 complex (significant decrease of phospho-S6 expression and no differences in phospho-AKT Ser473 expression) (Figure 2A,B). Torin2 treatment led to a significant and concurrent downregulation of mTORC1 and mTORC2 complex activity (significant decrease of phospho-S6 and phospho-AKT Ser473 expression) (Figure 2A,B). These effects were also observed after 60 h of treatment in both cell lines.

**Figure 3:**
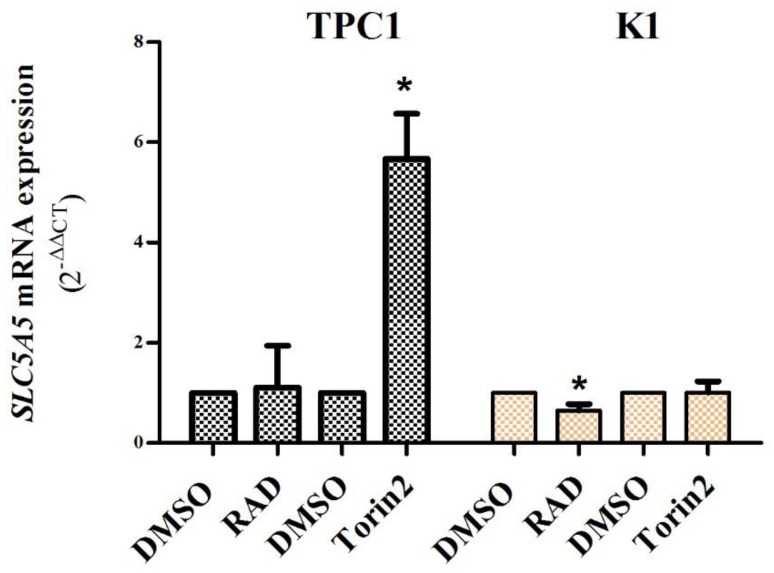
*SLC5A5* expression in TPC1 and K1 cell lines after treatment with RAD001 (20 nM) and Torin2 (450 nM) for 72 h. Mean fold change of *SLC5A5* mRNA expression observed in TPC1 and K1 cell lines after treatment in comparison to cells treated with DMSO. Treatment with RAD001 did not affect *SLC5A5* expression in the TPC1 cell line and caused a slight decrease in the K1 cell line. Treatment with Torin2 caused a significant increase (~6 fold) in *SLC5A5* expression in the TPC1 cell line but not in the K1 cell line. Bars represent mean expression ± SEM. * *p* < 0.05. Results are shown as mean expression values of three independent experiments.

At 72h, RAD001 treatment did not affect *SLC5A5* mRNA expression in the TPC1 cell line and caused a slight decrease in the K1 cell line. Torin2 treatment caused a significant increase in *SLC5A5* mRNA expression (~6 fold, *p* = 0.02) in the TPC-1 cell line but had no effect on the K1 cell line (Figure 3). *SLC5A5* mRNA expression was not altered in either cell line after 60 h of treatment with both drugs (except for a slight decrease of *SLC5A5* mRNA expression in the K1cell line after 60 h of treatment with RAD001).

### 2.4. BRAF Regulation of SLC5A5 mRNA Expression and of mTORC1 and mTORC2 Status in K1 Cell Line

**Figure 4:**
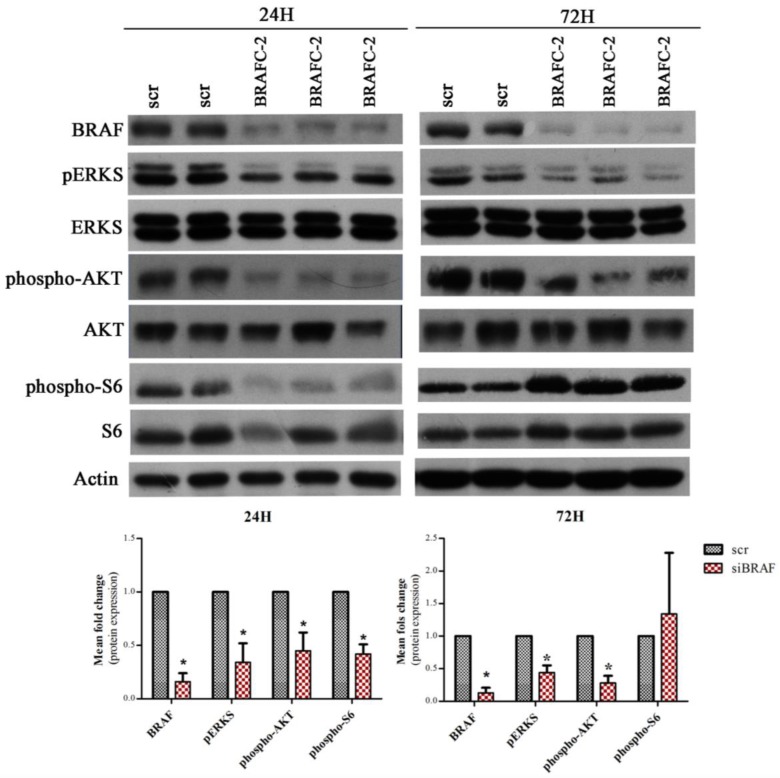
*BRAF*, pERKS, ERKS, phospho-AKT Ser473, AKT, phospho-S6 Ser235/236, and S6 expression in the K1 cell line after *BRAF* silencing. Western blot for *BRAF*, pERKS, ERKS, phospho-AKT Ser473, AKT, phospho-S6 Ser235/236, S6 expression and actin in K1 cell line treated with *BRAF*-C2 siRNA (50 nM) after 24 and 72 h. The levels of *BRAF* were analyzed for control of silencing efficiency and the levels of pERKS as a readout of MAPK pathway activity. Representative actin expression pattern is shown. Protein level, in scramble siRNA treated cells, was evaluated in duplicate (Scr), whereas in *BRAF* siRNA treated cells, it was analyzed in triplicate. The graphics depicts the mean fold change of protein expression observed in K1 cell line treated with *BRAF*-C2 siRNA in comparison to cells treated with scramble siRNA. Phosphorylated proteins were normalized by the levels of their correspondent total proteins, all others were normalized by the levels of control protein (actin). Results are shown as mean expression value of two independent experiments ±SEM. * *p* < 0.05 (unpaired Student’s t test).

**Figure 5:**
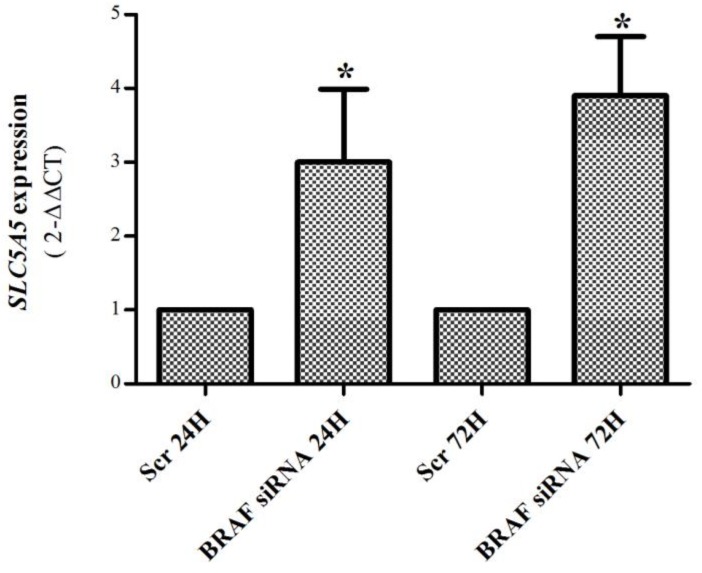
*SLC5A5* mRNA expression in K1 cell line after *BRAF* silencing (50 nM, 24 and 72 h). *BRAF* silencing caused a significant (~3 fold) increase in *SLC5A5* mRNA expression at both time points. Bars represent mean expression ± SEM. * *p* < 0.05. Results are shown as mean expression values of two independent experiments, each one with three replicates.

Given the lack of effect of Torin2 to increase *SLC5A5* mRNA expression in the K1 cell line, we hypothesized that it may be related to the fact that the K1 cell line presents the *BRAF*V600E mutation (known to decrease *SLC5A5* mRNA expression [23]). In order explore this hypothesis we performed *BRAF* down-regulation by siRNA (24 h and 72 h). As observed in Figure 4, *BRAF*-C2 siRNA led to a significant downregulation of *BRAF* and pERKs, confirming the downregulation of the MAPK pathway. *BRAF* silencing in the K1 cell line caused a significant increase in *SLC5A5* mRNA expression (~3 fold increase) (Figure 5). 

Since we observed, in our tumor samples, that PTCs harboring the *BRAF*V600E mutation presented higher levels of phospho-AKT Ser473, we were also interested in the effect of the *BRAF*V600E mutation in the activation status of each mTOR complex. To address this issue, we also evaluated phospho-AKT Ser473 and phospho-S6 expression (mTORC1 and mTORC2 readout, respectively) after *BRAF* silencing. After 24 h of *BRAF* silencing, both phospho-S6 Ser235/236 and phospho-AKT Ser473 expression were significantly decreased, however after 72 h of silencing, phospho-AKT Ser473 expression remained significantly lower while phospho-S6 Ser235/236 expression increased and became higher in silenced cells compared to scramble cells. 

## 3. Discussion

In a recent study by our group we demonstrated that phospho-mTOR is a marker of aggressiveness in PTC, as its expression is associated with aggressive clinicopathological features, including distant metastases, resistance to ^131^I therapy, and, consequently, worse prognosis [13]. Paradoxically, in the same study, we did not find a significant correlation between phospho-mTOR and phospho-S6 expression [13]. This led us to hypothesize that, in PTC, the activation of phospho-mTOR might be contributing preferentially to the activation of the mTORC2 complex, and consequently, to AKT phosphorylation (phospho-AKT Ser473) [13]. In the present study, we observed a positive and significant correlation between phospho-mTOR and phospho-AKT Ser473 expression, indicating that PTCs that expressed higher levels of phospho-mTOR also expressed higher levels of phospho-AKT Ser473. We also demonstrated that phospho-AKT Ser473 nuclear expression is associated with the presence of distant metastases. These results support our hypothesis that, in PTC, mTOR phosphorylation may lead to the preferential activation of the mTORC2 complex and its downstream effector phospho-AKT Ser473, which seems to play a role in distant metastization. 

Preferential formation of the mTORC2 complex was previously observed in other human malignancies and is usually associated with increased cell motility [14,15,16,17]. In TC, both mTORC1 and mTORC2 complexes are overexpressed compared to normal tissues [9,21], but the contribution of each complex to tumor behavior and prognosis still needs further investigation. Previous studies showed that phospho-AKT Ser473 is overexpressed in TC [9,11,12,24], and its expression has been associated with metastization in animal models of TC [25,26]. Recently, a study by Matson et al. [18] demonstrated that the depletion of AKT1 expression in TC cell lines was able to decrease invasion.

Our results indicate that the activation of phospho-AKT Ser473 plays a role in TC distant metastization. We observed that phospho-AKT Ser473 expression was associated with distant metastasis only when nuclear expression was considered. It seems that phospho-AKT Ser473 nuclear translocation is of major importance to migration and distant metastization of TC. Vasko et al. [12] demonstrated that phospho-AKT Ser473 was expressed in the cytoplasm of PTC throughout the tumor, but the immunostaining was more intense and localized in the nucleus of cells located in the invasive regions. We also observed that when phospho-AKT Ser473 staining was more concentrated in the invasive front of the tumor, it was preferentially located in the nucleus. Moreover, in an animal model of TC, phospho-AKT Ser473 expression was localized primarily in the nucleus of cells from metastatic lesions, while in the respective primary tumors it was located in the cytoplasm and nucleus of cells. These results suggest that phospho-AKT Ser473 nuclear distribution may be relevant for promoting metastization [26]. 

As previously mentioned, these results contrast with the lack of correlation between phospho-mTOR and phospho-S6 expression, as well as the distinct behavior associated with the expression of each marker observed in our previous study [13]. One possible interpretation is the fact that phospho-S6 can be phosphorylated (at Ser235/236) in an mTORC1 independent manner. For instance, S6K1-/-/2-/- knock-out mice were found to display no phosphorylation of phospho-S6 Ser240/244 but present persistent phosphorylation at phospho-S6 Ser235/236, revealing the presence of another in vivo phospho-S6 kinase, p90 ribosomal S6 kinase (RSK) [27], which can phosphorylate S6 in response to the RAS/ERK pathway, serum, and growth factors independently of mTORC1 [28]. Furthermore, in some situations, the equal activation of both mTOR complexes may not coexist, since it has already been shown that mTORC1 inhibition can increase mTORC2 activation. Inhibition of mTOR/S6K1 can induce phospho-AKT S473 phosphorylation (by a negative feedback loop) through the activation of insulin receptor substrate-1 (IRS1) function, a mediator of the insulin receptor-dependent activation of PI3K [29]. 

**Figure 6:**
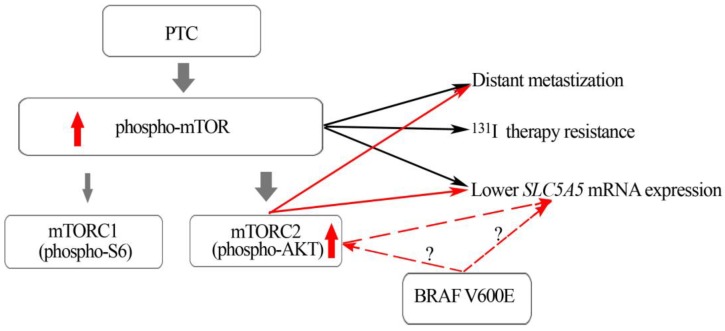
mTOR can be found in two distinct complexes: mTORC1 and mTORC2, each one with different downstream effectors, pS6 and pAKT, respectively. In PTCs, MTOR activation (high phosphor-mTOR expression) and the mTORC2 downstream effector (nuclear phospho-AKT Ser473) are associated with aggressive features (distant metastization). PTCs harboring *BRAF*V600E also present higher levels of pAKT. The impact of *BRAF* V600E on *SLC5A5* mRNA expression can be direct or mediated by activation of pAKT. The red dotted arrows refers to different possibilities.

We also found that phospho-AKT Ser473 expression (cytoplasmic and nuclear) was significantly higher in PTCs harboring the *BRAF*V600E mutation compared to *BRAF*WT PTCs (Table 2). In our previous study we observed that *BRAF*V600E mutated PTCs expressed similar high levels of phospho-mTOR but significantly lower levels of phospho-S6 compared to *BRAF*WT PTCs [13]. Altogether, the results of both studies suggest that PTCs harboring a *BRAF*V600E mutation display a preferential activation of the mTORC2 complex in comparison to mTORC1 (Figure 6). Our in vitro results reinforce this assumption: After 24 h of *BRAF* silencing we observed a significant decrease of phospho-S6 Ser235/236 and phospho-AKT Ser473 expression, nevertheless after 72 h, even though *BRAF* was still silenced and pERKS diminished, phospho-S6 Ser235/236 expression increased while phospho-AKT Ser 473 protein levels remained significantly decreased. These results suggest that *BRAF*V600E might have a long-lasting effect in the regulation of phospho-AKT Ser 473 expression (mTORC2) compared to phospho-S6 Ser235/236 expression (mTORC1). 

Recent studies explored the role of the mTOR pathway in NIS expression/function in rat thyroid cells [19] and in human TC cell lines (8505C, TPC1, and BCPAP) [20], demonstrating that treatments with rapamycin, a mTORC1 inhibitor, were able to restore NIS expression and function in some cell lines, but not in TPC-1 [19,20]. Loss of NIS expression and function has been indicated as the molecular mechanism responsible for radioactive iodine therapy resistance and metastatic progression in TC [7]. Since our results indicated preferential mTORC2 activation in PTCs, we have also become interested in exploring the role of mTORC2 in the NIS protein and *SLC5A5* mRNA expression. We performed our study in TPC1 and K1 cell lines. RAD001 caused a decrease in phospho-S6 expression, but it did not alter phospho-AKT Ser473 nor *SLC5A5* expression in both cell lines (as it was already observed for the TPC1 cell line) [20]. On the other hand, Torin2 treatment caused a decrease of phospho-S6 and phospho-AKT Ser473 expression in both cell lines, and a significant increase in *SLC5A5* mRNA expression, but only in the TPC1 cell line (Figure 2 and Figure 3). These results demonstrate that the inhibition of the mTORC2 complex may be of major importance in the restoration of *SLC5A5* mRNA expression, highlighting its role as a potential therapeutic target. To the best of our knowledge, the impact of Torin2 in *SLC5A5* mRNA expression or NIS protein function has not been previously addressed. Of note, patients with PTC that developed recurrences and/or distant metastases presented lower levels of *SLC5A5* mRNA expression compared to patients without tumor progression [30]. This information is in line with our present results indicating that mTORC2 (phospho-AKT Ser 473) can be implicated in distant metastization as well as in the regulation of *SLC5A5* mRNA expression.

The different responses to Torin2 treatment, in terms of *SLC5A5* mRNA expression, of the two cell lines, led us to focus on the differences between them. One major difference is the genetic background: the TPC1 cell line harbors rearranged during transfection proto-oncogene *RET/PTC*1 rearrangement, while the K1 cell line harbors the *BRAF*V600E point mutation [31]. It is well established that, at variance to *RET/PTC* rearrangement, *BRAF*V600E mutation impairs *SLC5A5* mRNA expression, as well as NIS trafficking to the basolateral membrane in patients and cell lines [23,32]. The molecular mechanism behind this impairment is not fully understood yet, and even though the *BRAF*V600E mutation activates the MAPK pathway, its effect on NIS impairment does not seem to be mediated by the MAPK pathway [23]. Taking this information into consideration, we performed *BRAF* silencing in the K1 cell line to evaluate if *BRAF* was interfering with *SLC5A5* mRNA expression. In fact, after *BRAF* silencing, we observed a significant increase in *SLC5A5* mRNA expression, as well as a decrease in phosphor AKT Ser 473 expression. Gathering the literature [23,32] together with our present results, we hypothesize that in a *BRAF*V600E context, the concurrent mTORC1 and mTORC2 downregulation may not be sufficient to induce *SLC5A5* mRNA expression, thus explaining the absence of an increase in *SLC5A5* mRNA expression in the K1cell line after Torin2 treatment. 

Summing up, we have demonstrated that the mTORC2 pathway is activated in PTC, especially in those PTC harboring the *BRAF*V600E mutation. We have also shown that in the mTORC2 downstream effector phospho-AKT Ser473, nuclear translocation may play a role in distant metastization, and, possibly, in *SLC5A5* mRNA downregulation. We propose that, in PTC, the mTORC2 complex may be preferentially activated (phospho-AKT473), and that this specific complex may be implicated in the distant metastization, decrease in *SLC5A5* mRNA expression, and therapy resistance.

## 4. Materials and Methods

### 4.1. Patient Tissue Samples 

One hundred and eighty-two formalin-fixed, paraffin-embedded representative tissue samples of PTCs were collected from files of the Institute of Molecular Pathology and Immunology of the University of Porto (IPATIMUP, Porto, Portugal), corresponding to 182 patients followed in two university hospitals in Portugal. In 115 cases we had access to follow-up data. The histology of all tumors samples was revised (CE, ER, MSS) according to the World Health Organization criteria [33]. Epidemiological, clinical, and molecular data of the 182 cases are summarized in Table 4. This work was approved by the Ethic Committee for Health (CES) of the Hospital Center of São João (CHSJ)/Faculty of Medicine of the University of Porto (FMUP) (CES 137 284-13, 2014) and by the Ethics Committee of the Faculty of Medicine of the University of Coimbra (n º1309, 2010). All procedures described in this study were in accordance with National Ethical Standards (Law nº 12/2005) and the Helsinki Declaration. 

### 4.2. Patient’s Follow Up

Patients were treated and followed in accordance with the international protocols available at the time. Data regarding the number of radioiodine treatments and cumulative activity were retrieved from hospital records. Patients were considered as being disease free at the end of follow-up if they had undetectable stimulated thyroglobulin (in the absence of thyroglobulin antibodies) and no imagiological evidence of the disease. The mean time of follow up was 8.0 ± 6.8 years. The number of ^131^I treatments varied between 1 and 5 (mean 1.8) and cumulative dose of RAI totalized values were between 30 and 1146 mCi (mean 245.2 mCi). For statistical analysis, we defined the category “additional treatments”, in which we included other treatment modalities in addition to radioiodine, including extra surgery, external beam irradiation, and treatment with tyrosine kinase inhibitors.

### 4.3. Immunohistochemistry

Immunohistochemistry was performed as previously described [9]. Briefly, sections were subjected to heat-induced antigen retrieval in 10 mM sodium citrate buffer (pH 6.0). Endogenous peroxidase activity was blocked with 3% of H_2_O_2_ and nonspecific binding with Large Volume Ultra V Block reagent (Thermo Scientific/Lab Vision, Waltham, MA, USA). Sections were then incubated overnight at 4 °C with anti-phospho-AKT Ser473 antibody (clone 736E11) (Cell Signaling Technology, Danvers, MA, USA) (1:50). The detection was performed with a labeled streptavidin-biotin immunoperoxidase detection system (Thermo Scientific/Lab Vision, Waltham, MA, USA) followed by 3,3′-diaminobenzidine (Dako, Glostrup, Denmark) reaction and counterstained with hematoxylin.

The immunostaining evaluation was done according to our previous work [9]. Slides were evaluated by two independent observers and semiquantitatively scored in terms of percentage of tumor stained cells (0: ˂5%; 1: 5–25%; 2: 25–50%; 3: 50–75%; 4: ˃75%) and staining intensity (0—negative; 1—weak; 2—intermediate; 3—strong). An immunohistochemical score was calculated by multiplying the proportion of positive cells by the intensity of the staining, 12 being the maximum score. The distribution of cases within the scores is summarized in Table 4. The cellular localization was also evaluated as membrane and/or cytoplasmic and/or nuclear. To be considered positive for nuclear expression, tumors must display phospho-AKT Ser473 immunostaining in at least 5% of tumor cells. As a positive control, we used a breast carcinoma known to be positive for phospho-AKT Ser473, for a negative control, we used the same carcinoma and omitted the primary antibody (Figure S1). Slides were observed using a Axioskop 2 Zeiss microscope. Representative slides were scanned using a DSight Viewer (Menarini, Florence, Italy) and photographs were obtained through snapshots from DSight Viewer Software (Menarini). From the 182 cases characterized for phospho-AKT Ser473, 170 have been previously characterized for phospho-mTOR Ser2448 and 141 for phospho-S6 Ser235/236 [13].

### 4.4. DNA Extraction, PCR and Sanger Sequencing

The genetic characterization (PCR and sequencing) of the tumors regarding *BRAF*, *NRAS*, *RET/PTC*, and *TERT* promoter (*TERTp*) mutations were screened as previously described [34,35,36,37,38], and in part as had been previously reported [13]. 

### 4.5. Cell Lines, Treatments with RAD001 and Torin2 and Transfection Assays

The TPC1 cell line was kindly provided by Doctor Dumont JE and Doctor Marell M, and the K1 cell line was provided by Dr. Wynford-Thomas D [31]. Both cell lines were derived from papillary thyroid carcinoma. They have already been characterized at the molecular and genotypic level, TPC1 cell line harbors the *RET/PTC*1 rearrangement and *TERT*p mutation (−124 G>A). The K1 cell line harbors the *BRAF*V600E and *PI3K*E542K mutations and also the *TERT*p mutation (−124 G>A) [31,38]. Cell lines were maintained in RPMI supplemented with antibiotics; 1% (*v*/*v*) Pen Strep and 0.5% fungizone (*v*/*v*) (Biowest, Nuaillé, France) and 10% (*v*/*v*) of fetal bovine serum (FBS) (GIBCO, Thermo Fisher Scientific Waltham, MA, USA). Cells were grown in a humidified incubator with 5% CO_2_ at 37 °C. 

For treatment purposes, cell were plated in six well plates, TPC1 (1 × 10^5^ cells per well) and K1 (2 × 10^5^ cells per well), 24 h later cells were treated with RAD001 (20 nM) or Torin2 (450 nM) (Selleckchem, Houston, TX, USA). Treatments lasted for 60 h and 72 h. Treatments were performed in triplicate, each experiment had two replicates of the treatment. 

Small interfering RNAS (siRNA) assays were performed as previously reported [39], using 50 nM of oligo *BRAF* (BRAF-C2), cell lysates were obtained after 24 h and 72 h. Silencing was performed in duplicate (two independent experiments), each experiment had two replicates of the scramble and tree replicates of the silencing. 

### 4.6. RNA Extraction, Reverse Transcription and Real Time PCR

Total RNA was extracted from TPC1 and K1 cell lines using a Trizol commercial kit (Thermo Scientific/GIBCO, Waltham, MA, USA) according to the manufacturer’s protocol. RNA was quantified by spectrophotometry, and its quality was checked by analysis of 260/280 nm and 260/230 nm ratios. For cDNA preparation, 1 μg of total RNA was reverse transcribed using the RevertAid first strand cDNA synthesis kit (Thermo Scientific/Fermentas, Waltham, MA, USA). 

Reverse transcription products were amplified for *SLC5A5* by qPCR (IDT:Integrated DNA Technologies, Leuven, Belgium; no. HS.PT.56a.40789288) using a TaqMan PCR Master Mix (Applied Biosystems, Foster City, CA, USA) with the TBP gene (TATA-binding protein) as an endogenous control (Applied Biosystems; no. 4326322E-0705006). The ABI PRISM 7500 Fast Sequence Detection System (Applied Biosystems, Foster City, CA, USA) was used and was programmed to an initial step of 20 s at 50 °C, 10 min at 95° C, followed by 40 cycles of 95 °C for 15 s and 60 °C for 1 min. For each sample, TBP and *SLC5A5* amplifications were done in triplicate using 1 μL of cDNA (~25 ng). 

The relative quantification of target genes was determined using the 2^−ΔΔ^CT method. Similar efficiencies of both assays were confirmed using Livak’s Linear Regression Method [40] (slope −0.4). 

### 4.7. Western Blot Analysis

Cells were lysed in RIPA buffer supplemented with phosphatase and protease inhibitors. Proteins were quantified using DC™ Protein Assay (Bio-RAD, CA, USA), then were resolved by SDS-PAGE and transferred onto nitrocellulose membranes (GE Healthcare, Little Chalfont, UK). The primary antibodies were: phospho-S6 Ser235/236, S6, phospho-AKTSer473, AKT, pERKS, ERKS (1:1000), and *BRAF* (1:500) (Santa Cruz Biotechnology, Santa Cruz, CA, USA), all antibodies were acquired from Cell Signaling Technology (Danvers, MA, USA).

Protein was detected using a horseradish peroxidase-conjugated antibody (Santa Cruz Biotechnology, Santa Cruz, CA, USA) and a luminescence system (Perkin-Elmer, Waltham, MA, USA). For the protein loading control, membranes were incubated with an antiactin Santa Cruz Biotechnology (Santa Cruz, CA, USA) antibody. Protein expression was quantified using the Bio-Rad Quantity One 1-D Analysis software (Bio-Rad Laboratories, Inc., Hercules, CA, USA). The levels of phosphorylated proteins: phospho-S6 Ser235/236, phospho-AKT Ser 473, and pERKS were normalized by the levels of their corresponding total protein (total, S6, and AKT), all others were normalized by loading control (actin). The levels of expression of phosphorylated proteins and their corresponding total protein were evaluated in the same gel, furthermore, the antibodies used for the total proteins recognize all forms of the phosphorylated proteins. 

### 4.8. Statistical Analysis

Statistical analysis was conducted with SPSS version 21.00 (SPSS Inc). The expression of phospho-AKT Ser473 is expressed as mean ± standard deviation. An independent sample Student’s t test was used to evaluate possible associations between phospho-AKT Ser 473 expression and clinicopathological and molecular features to compare protein expression (analyzed by western blot) between groups. A Pearson Correlation was used to evaluate the correlation between phospho-AKT Ser473, phospho-mTOR Ser2448, and phospho-S6 Ser235/236 expression. A Chi-square test was used to evaluate possible associations between phospho-AKT Ser 473 nuclear expression and clinicopathological and molecular features. Results were considered statistically significant at *p ≤* 0.05.

## Figures and Tables

**Table 1:** Distribution of phospho-AKT score throughout the series.

Phospho-AKT Score	Frequency	%
0	90	49.5
1	18	9.9
2	15	8.2
3	6	3.3
4	8	4.4
6	14	7.7
8	11	6.0
9	6	3.3
12	14	7.7
Total	182	100

**Table 2:** Association between phospho-AKT score and *BRAF* status.

*BRAF*	Phospho-AKT Score	*p* Value
WT (*n* = 106)	2.2 ± 3.3	0.04
V600E (*n* = 74)	3.4 ± 4.4	

WT: wild type

**Table 3:** Association between phospho-AKT nuclear expression and distant metastases.

Nuclear Expression	Distant Metastases		*p* Value
	Yes	No	
Yes	9 (81.82%)	19 (47.5%)	
No	2 (18.18%)	21 (52.5%)	0.04
Total	11	40	51

**Table 4:** Epidemiologic, histologic, and clinical data of the patients.

Patients		Total and %		
		cPTC	fvPTC	other PTC variants
**Gender**	F *n* = 150	94 (82.5)	41 (87.2)	15 (71.4)
	M *n* = 32	20 (17.5)	6 (12.8)	6 (28.6)
**Age**	<45 years *n* = 94	62 (54.9)	21 (45.7)	11 (55.0)
	≥45 years *n* = 85	51 (45.1)	25 (54.3)	9 (45.0)
**Tumor size**	<2cm *n* = 64	39 (36.8)	17 (37.0)	8 (40.0)
	≥2cm *n* = 108	67 (63.2)	29 (63.0)	12 (60.0)
**Tumor capsule**	Present *n* = 83	42 (39.6)	32 (71.1)	9 (42.9)
	Absent *n* = 89	64 (60.4)	13 (28.9)	12 (57.1)
**Tumor capsule Invasion**	Yes *n* = 64	35 (89.7)	22 (68.8)	7 (100)
	No *n* = 14	4 (10.3)	10 (31.3)	0 (0)
**Extra thyroid invasion**	Yes *n* = 73	50 (48.1)	12 (27.3)	11 (55.0)
	No *n* = 95	54 (51.9)	32 (72.7)	9 (45.0)
**Multifocality**	Single *n* = 104	58 (54.7)	32 (68.1)	14 (70.0)
	Multiple *n* = 69	48 (45.3)	15 (31.9)	6 (30.0)
**Lymphocytic infiltrate**	Present *n* = 108	77 (70.6)	19 (41.3)	12 (60.0)
	Absent *n* = 67	32 (29.4)	27 (58.7)	8 (40.0)
**Vascular invasion**	Present *n* = 59	42 (40.4)	10 (22.2)	7 (35.0)
	Absent *n* = 110	62 (59.6)	35 (77.8)	13 (65.0)
**Lymph node metastases**	Present *n* = 57	40 (43.0)	12 (34.3)	5 (29.4)
	Absent *n* = 88	53 (57.0)	23 (65.7)	12 (70.6)
**Tumor margins**	Infiltrative *n* = 78	57 (79.2)	13 (46.4)	8 (72.7)
	Well defined *n* = 33	15 (20.8)	15 (53.6)	3 (27.3)
**Distant metastases**	Yes *n* = 17	9 (11.8)	5 (17.9)	3 (30.0)
	No *n* = 97	67 (88.2)	23 (82.1)	7 (70.0)
**One year disease free survival**	Yes *n* = 64	41 (56.2)	19 (67.9)	4 (40.0)
	No *n* = 47	32 (43.8)	9 (32.1)	6 (60.0)
**Disease free (at the end of follow up)**	Yes *n* = 70	44 (59.5)	19 (67.9)	7 (70.0)
	No *n* = 42	30 (40.5)	9 (32.1)	3 (30.0)
**Deaths**	Yes *n* = 5	2 (2.6)	2 (7.1)	1 (9.1)
	No *n* = 110	74 (97.4)	26 (92.9)	10 (90.9)
***BRAF***	WT *n* = 106	56 (49.1)	37 (82.2)	13 (61.9)
	V600E *n* = 74	58 (50.9)	8 (17.8)	8 (38.1)
***NRAS***	WT *n* = 162	108 (99.1)	38 (90.5)	16 (80.0)
	Mut *n* = 9	1 (0.9)	4 (9.5)	4 (20.0)
***TERTp***	WT *n* = 152	95 (96.0)	40 (95.2)	17 (100.0)
	Mut *n* = 6	4 (4.0)	2 (4.8)	0 (0.0)
***RET/PTC***	WT *n* = 56	29 (78.4)	18 (94.7)	9 (90.0)
	Rearrangment *n* = 10	8 (21.6)	1 (5.3)	1 (10.0)
**Staging**	I *n* = 64	45 (64.3)	15 (60.0)	4 (50.0)
	II *n* = 6	3 (4.3)	3 (12.0)	0 (0.0)
	III *n* = 24	19 (27.1)	3 (12.0)	2 (25.0)
	IV *n* = 9	3 (4.3)	4 (16.0)	2 (25.0)

## Supplementary Materials

Supplementary materials can be found at http://www.mdpi.com/1422-0067/19/5/1448/s1.

## Abbreviations

TC	thyroid cancer
PTC	papillary thyroid carcinoma
*SLC5A5*	Solute Carrier Family 5 Member 5
cPTC	classic PTC
fvPTC	follicular variant of PTC
NIS	sodium iodide symporter
RAI	radioactive iodine

## References

[1] Sipos J.A., Mazzaferri E.L. (2010). Thyroid cancer epidemiology and prognostic variables. Clin. Oncol..

[2] Petrulea M.S., Plantinga T.S., Smit J.W., Georgescu C.E., Netea-Maier R.T. (2015). PI3K/Akt/mTOR: A promising therapeutic target for non-medullary thyroid carcinoma. Cancer Treat. Rev..

[3] LiVolsi V.A. (2011). Papillary thyroid carcinoma: An update. Mod. Pathol..

[4] Soares P., Celestino R., Melo M., Fonseca E., Sobrinho-Simoes M. (2014). Prognostic biomarkers in thyroid cancer. Virchows Archiv Int. J. Pathol..

[5] Durante C., Haddy N., Baudin E., Leboulleux S., Hartl D., Travagli J.P., Caillou B., Ricard M., Lumbroso J.D., De Vathaire F. (2006). Long-term outcome of 444 patients with distant metastases from papillary and follicular thyroid carcinoma: Benefits and limits of radioiodine therapy. J. Clin. Endocrinol. Metab..

[6] Fallahi P., Mazzi V., Vita R., Ferrari S.M., Materazzi G., Galleri D., Benvenga S., Miccoli P., Antonelli A. (2015). New therapies for dedifferentiated papillary thyroid cancer. Int. J. Mol. Sci..

[7] Vaisman F., Carvalho D.P., Vaisman M. (2015). A new appraisal of iodine refractory thyroid cancer. Endocr.-Relat. Cancer.

[8] De Souza E.C.L., Freitas Ferreira A.C., de Carvalho D.P. (2011). The mTOR protein as a target in thyroid cancer. Expert Opin. Ther. Targets.

[9] Faustino A., Couto J.P., Populo H., Rocha A.S., Pardal F., Cameselle-Teijeiro J.M., Lopes J.M., Sobrinho-Simoes M., Soares P. (2012). mTOR pathway overactivation in BRAF mutated papillary thyroid carcinoma. J. Clin. Endocrinol. Metab..

[10] Populo H., Lopes J.M., Soares P. (2012). The mTOR signalling pathway in human cancer. Int. J. Mol. Sci..

[11] Miyakawa M., Tsushimma T., Murakami H., Wakai K., Isozaki O., Takano K. (2003). Increased expression of phosphorylated p70S6 kinase and Akt in papillary thyroid cancer tissues. Endocr. J..

[12] Vasko V., Saji M., Hardy E., Kruhlak M., Larin A., Savchenko V., Miyakawa M., Isozaki O., Murakami H., Tsushima T. (2004). Akt activation and localisation correlate with tumour invasion and oncogene expression in thyroid cancer. J. Med. Genet..

[13] Tavares C., Coelho M.J., Melo M., da Rocha A.G., Pestana A., Batista R., Salgado C., Eloy C., Ferreira L., Rios E. (2016). pmTOR is a marker of aggressiveness in papillary thyroid carcinomas. Surgery.

[14] Gupta S., Hau A.M., Beach J.R., Harwalker J., Mantuano E., Gonias S.L., Egelhoff T.T., Hansel D.E. (2013). Mammalian target of rapamycin complex 2 (mTORC2) is a critical determinant of bladder cancer invasion. PLoS ONE.

[15] Masri J., Bernath A., Martin J., Jo O.D., Vartanian R., Funk A., Gera J. (2007). mTORC2 activity is elevated in gliomas and promotes growth and cell motility via overexpression of rictor. Cancer Res..

[16] Bian Y., Wang Z., Xu J., Zhao W., Cao H., Zhang Z. (2015). Elevated Rictor expression is associated with tumor progression and poor prognosis in patients with gastric cancer. Biochem. Biophys. Res. Commun..

[17] Maru S., Ishigaki Y., Shinohara N., Takata T., Tomosugi N., Nonomura K. (2013). Inhibition of mTORC2 but not mTORC1 up-regulates E-cadherin expression and inhibits cell motility by blocking HIF-2α expression in human renal cell carcinoma. J. Urol..

[18] Matson D.R., Hardin H., Buehler D., Lloyd R.V. (2017). AKT activity is elevated in aggressive thyroid neoplasms where it promotes proliferation and invasion. Exp. Mol. Pathol..

[19] De Souza E.C., Padron A.S., Braga W.M., de Andrade B.M., Vaisman M., Nasciutti L.E., Ferreira A.C., de Carvalho D.P. (2010). MTOR downregulates iodide uptake in thyrocytes. J. Endocrinol..

[20] Plantinga T.S., Heinhuis B., Gerrits D., Netea M.G., Joosten L.A., Hermus A.R., Oyen W.J., Schweppe R.E., Haugen B.R., Boerman O.C. (2014). mTOR Inhibition promotes TTF1-dependent redifferentiation and restores iodine uptake in thyroid carcinoma cell lines. J. Clin. Endocrinol. Metab..

[21] Ahmed M., Hussain A.R., Bavi P., Ahmed S.O., Al Sobhi S.S., Al-Dayel F., Uddin S., Al-Kuraya K.S. (2014). High prevalence of mTOR complex activity can be targeted using Torin2 in papillary thyroid carcinoma. Carcinogenesis.

[22] Sadowski S.M., Boufragech M., Zhang L., Mehta A., Kapur P., Zhang Y., Li Z., Shen M., Kebebew E. (2015). Torin2 targets dysregulated pathways in anaplastic thyroid cancer and inhibits tumor growth and metastasis. Oncotarget.

[23] Riesco-Eizaguirre G., Gutierrez-Martinez P., Garcia-Cabezas M.A., Nistal M., Santisteban P. (2006). The oncogene BRAF V600E is associated with a high risk of recurrence and less differentiated papillary thyroid carcinoma due to the impairment of Na+/I− targeting to the membrane. Endocr.-Relat. Cancer.

[24] Ringel M.D., Hayre N., Saito J., Saunier B., Schuppert F., Burch H., Bernet V., Burman K.D., Kohn L.D., Saji M. (2001). Overexpression and overactivation of Akt in thyroid carcinoma. Cancer Res..

[25] Saji M., Narahara K., McCarty S.K., Vasko V.V., la Perle K.M., Porter K., Jarjoura D., Cheng S.-Y., Lu C., Ringel M.D. (2011). Akt deficiency delays tumor progression, vascular invasion, and distant metastases in a murine model of thyroid cancer. Oncogene.

[26] Kim C.S., Vasko V.V., Kato Y., Kruhlak M., Saji M., Cheng S.Y., Ringel M.D. (2005). AKT activation promotes metastasis in a mouse model of follicular thyroid carcinoma. Endocrinology.

[27] Pende M., Um S.H., Mieulet V., Sticker M., Goss V.L., Mestan J., Mueller M., Fumagalli S., Kozma S.C., Thomas G. (2004). S6K1−/−/S6K2−/− Mice Exhibit Perinatal Lethality and Rapamycin-Sensitive 5′-Terminal Oligopyrimidine mRNA Translation and Reveal a Mitogen-Activated Protein Kinase-Dependent S6 Kinase Pathway. Mol. Cell Biol..

[28] Roux P.P., Shahbazian D., Vu H., Holz M.K., Cohen M.S., Taunton J., Sonenberg N., Blenis J. (2007). RAS/ERK signaling promotes site-specific ribosomal protein S6 phosphorylation via RSK and stimulates cap-dependent translation. J. Biol. Chem..

[29] Breuleux M., Klopfenstein M., Stephan C., Doughty C.A., Barys L., Maira S.-M., Kwiatkowski D., Lane H.A. (2009). Increased AKT S473 phosphorylation after mTORC1 inhibition is rictor dependent and does not predict tumor cell response to PI3K/mTOR inhibition. Mol. Cancer Ther..

[30] Tavares C., Coelho M.J., Eloy C., Melo M., da Rocha A.G., Pestana A., Batista R., Ferreira L.B., Rios E., Selmi-Ruby S. (2018). NIS expression in thyroid tumors, relation with prognosis clinicopathological and molecular features. Endocr. Connect..

[31] Meireles A.M., Preto A., Rocha A.S., Rebocho A.P., Maximo V., Pereira-Castro I., Moreira S., Feijao T., Botelho T., Marques R. (2007). Molecular and genotypic characterization of human thyroid follicular cell carcinoma-derived cell lines. Thyroid Off. J. Am. Thyroid Assoc..

[32] Durante C., Puxeddu E., Ferretti E., Morisi R., Moretti S., Bruno R., Barbi F., Avenia N., Scipioni A., Verrienti A. (2007). BRAF mutations in papillary thyroid carcinomas inhibit genes involved in iodine metabolism. J. Clin. Endocrinol. Metab..

[33] DeLellis R.A., Lloyd R.V., Heitz P.U., Eng C., WHO Classification of Tumours (2004). Pathology and Genetics of Tumours of Endocrine Organs.

[34] Soares P., Trovisco V., Rocha A.S., Lima J., Castro P., Preto A., Maximo V., Botelho T., Seruca R., Sobrinho-Simoes M. (2003). BRAF mutations and RET/PTC rearrangements are alternative events in the etiopathogenesis of PTC. Oncogene.

[35] Trovisco V., Soares P., Preto A., de Castro I.V., Lima J., Castro P., Maximo V., Botelho T., Moreira S., Meireles A.M. (2005). Type and prevalence of BRAF mutations are closely associated with papillary thyroid carcinoma histotype and patients’ age but not with tumour aggressiveness. Virchows Archiv Int. J. Pathol..

[36] De Vries M.M., Celestino R., Castro P., Eloy C., Maximo V., van der Wal J.E., Plukker J.T., Links T.P., Hofstra R.M., Sobrinho-Simoes M. (2012). RET/PTC rearrangement is prevalent in follicular Hurthle cell carcinomas. Histopathology.

[37] Melo M., da Rocha A.G., Vinagre J., Batista R., Peixoto J., Tavares C., Celestino R., Almeida A., Salgado C., Eloy C. (2014). TERT promoter mutations are a major indicator of poor outcome in differentiated thyroid carcinomas. J. Clin. Endocrinol. Metab..

[38] Vinagre J., Almeida A., Populo H., Batista R., Lyra J., Pinto V., Coelho R., Celestino R., Prazeres H., Lima L. (2013). Frequency of TERT promoter mutations in human cancers. Nat. Commun..

[39] Preto A., Goncalves J., Rebocho A.P., Figueiredo J., Meireles A.M., Rocha A.S., Vasconcelos H.M., Seca H., Seruca R., Soares P. (2009). Proliferation and survival molecules implicated in the inhibition of BRAF pathway in thyroid cancer cells harbouring different genetic mutations. BMC Cancer.

[40] Livak K.J., Schmittgen T.D. (2001). Analysis of relative gene expression data using real-time quantitative PCR and the 2(-Delta Delta C(T)) Method. Methods.

