# Supplementary material for: mTOR Pathway in Papillary Thyroid Carcinoma: Different Contributions of mTORC1 and mTORC2 Complexes for Tumor Behavior and SLC5A5 mRNA Expression
Source: Int J Mol Sci. 2018 May 13;19(5):1448. doi: 10.3390/ijms19051448 (PMC5983778; doi:10.3390/ijms19051448)
Supplement: Supplementary file 1 [file ijms-19-01448-s001.pdf]

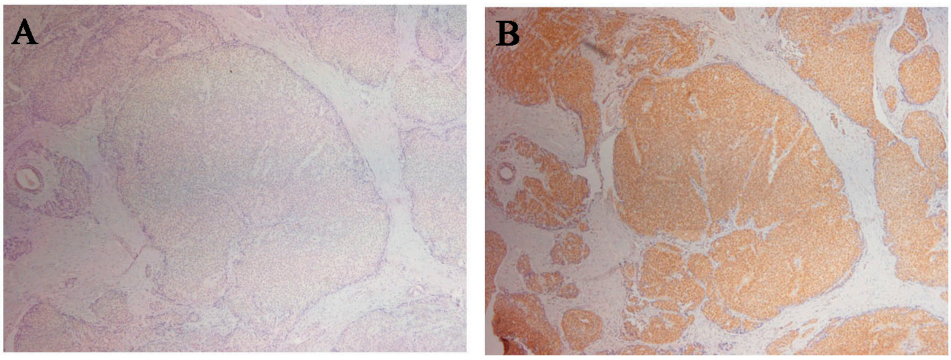

Figure S1. Breast carcinoma used as negative and positive control for phospho-AKT Ser473 immunostaining. (**A**) Negative control (omitting primary antibody); (**B**) Positive control. 5× magnification.
